# Neural Constraints Affect the Ability to Generate Hip Abduction Torques When Combined With Hip Extension or Ankle Plantarflexion in Chronic Hemiparetic Stroke

**DOI:** 10.3389/fneur.2018.00564

**Published:** 2018-07-11

**Authors:** Natalia Sánchez, Ana M. Acosta, Roberto López-Rosado, Julius P. A. Dewald

**Affiliations:** ^1^Division of Biokinesiology and Physical Therapy, University of Southern California, Los Angeles, CA, United States; ^2^Department of Physical Therapy and Human Movement Sciences, Northwestern University, Chicago, IL, United States; ^3^Department of Biomedical Engineering, Northwestern University, Chicago, IL, United States; ^4^Department of Physical Medicine and Rehabilitation, Northwestern University, Chicago, IL, United States

**Keywords:** stroke, joint torque, coupling, impairment, lower extremity, voluntary, abduction, extension

## Abstract

Stroke lesions interrupt descending corticofugal fibers that provide the volitional control of the upper and lower extremities. Despite the evident manifestation of movement impairments post-stroke during standing and gait, neural constraints in the ability to generate joint torque combinations in the lower extremities are not yet well determined. Twelve chronic hemiparetic participants and 8 age-matched control individuals participated in the present study. In an isometric setup, participants were instructed to combine submaximal hip extension or ankle plantarflexion torques with maximal hip abduction torques. Statistical analyses were run using linear mixed effects models. Results for the protocol combining hip extension and abduction indicate that participants post-stroke have severe limitations in the amount of hip abduction torque they can generate, dependent upon hip extension torque magnitude. These effects are manifested in the paretic extremity by the appearance of hip adduction torques instead of hip abduction at higher levels of hip extension. In the non-paretic extremity, significant reductions of hip abduction were also observed. In contrast, healthy control individuals were capable of combining varied levels of hip extension with maximal hip abduction. When combining ankle plantarflexion and hip abduction, only the paretic extremity showed reductions in the ability to generate hip abduction torques at increased levels of ankle plantarflexion. Our results provide insight into the neural mechanisms controlling the lower extremity post-stroke, supporting previously hypothesized increased reliance on postural brainstem motor pathways. These pathways have a greater dominance in the control of proximal joints (hip) compared to distal joints (ankle) and lead to synergistic activation of musculature due to their diffuse, bilateral connections at multiple spinal cord levels. We measured, for the first time, bilateral constraints in hip extension/abduction coupling in hemiparetic stroke, again in agreement with the expected increased reliance on bilateral brainstem motor pathways. Understanding of these neural constraints in the post-stroke lower extremities is key in the development of more effective rehabilitation interventions that target abnormal joint torque coupling patterns.

## Introduction

Stroke affects 6.6 million people in the United States with about 800,000 new and recurring strokes occurring every year (1). Stroke-induced brain injury interrupts descending motor pathways affecting motor commands from the cortex to motor neurons innervating the extremities. Despite the disruption of motor commands to the lower extremity, over 80% of stroke survivors regain the ability to stand and walk (2). However, stance, balance and gait post-stroke differ significantly from healthy individuals: upright stance is asymmetrical and biased toward the non-paretic leg (3, 4). Likewise, post-stroke gait is asymmetric (5–9), slow (7, 10–12) and prone to falls (13–16). These differences may be in part due to changes in the voluntary control of the lower extremity which may constrain joint torque combinations, leading individuals to over-rely on their non-paretic leg. Nonetheless, existence of constraints in the voluntary control of lower extremities after stroke and their potential impact during upright function are yet to be fully understood.

After stroke, interruption of corticofugal pathways has been hypothesized to result in an increased reliance on diffuse brainstem pathways that branch across multiple spinal segments (17–19). These diffuse pathways activate multiple motor neuron pools and consequently, multiple muscles simultaneously, which in the upper extremity, leads to abnormal muscle coactivation (20) and loss of independent joint control due to an increased dependence on contralesional corticoreticulospinal pathways (20–24). Similarly, in the lower extremity, previous studies have shown that independent joint control might be affected when generating maximal and submaximal voluntary torques in either hip extension or ankle plantarflexion (25). Generation of maximal ankle plantarflexion and maximal hip extension in individuals post-stroke leads to coupled extension/adduction torques across the hip, knee and ankle joints (25–27). These results are in agreement with what is referred clinically as the extensor synergy: coupling of hip extension and hip adduction with knee extension and ankle plantarflexion (28, 29). Despite the apparent dominance of the extensor synergy, research has yet to demonstrate whether individuals post-stroke can generate lower extremity joint torque combination patterns away from this synergy.

In this study, we probed the ability of participants to generate joint torque couples outside of the extensor synergy, by assessing their capacity to concurrently generate hip abduction torques with hip extension or ankle plantarflexion torques. We also investigated whether instructing participants to couple hip flexion with hip abduction, i.e., coupling inside the flexor synergy, facilitated the generation of hip abduction torque, which is significantly weakened after stroke (25), thus providing insight into the potential functional importance of the flexor synergy (28). We hypothesized that given the changes in the neural control of the lower extremity post-stroke, attributed to increased reliance on bilateral brainstem pathways that control proximal and postural hip muscles (30–32), the capacity to generate hip abduction with both the paretic and non-paretic extremities would be constrained by hip extension but would possibly benefit from hip flexion. In contrast, in control participants, we expected that the magnitude of hip abduction torques would be independent from the torques generated in the hip flexion/extension degree of freedom. The present characterization is of critical importance because it may help explain the impairments seen during upright posture both in quiet standing (3, 33) and in gait (10, 11, 34). For instance, the inability to couple hip extension with abduction may explain the failure to stabilize the pelvis leading to pelvic drop during single leg stance (11). Also, restrictions in hip abduction torque generation may hinder frontal plane displacement of the center of mass (35), compromising the ability to generate adequate responses to medio-lateral perturbations during standing (15). A better understanding of constraints in the ability to generate hip abduction during hip extension or ankle plantarflexion in the lower extremity after stroke may lead to the development of more targeted rehabilitation interventions that seek to restore individual joint control and coordination and are expected to improve standing posture and balance in people post-stroke.

## Methods

### Participants

Participants post-stroke were recruited for this study from the Clinical Neuroscience Research Registry, and the study was approved by Northwestern University's institutional review board. Initial evaluation of motor impairment was done using the lower extremity portion of the Fugl-Meyer (F-M) Motor Assessment (36). The Berg Balance Test (37) and the 10 m walk test (38) were used to assess balance and walking speed respectively.

All participants post-stroke had a unilateral brain lesion from a single stroke at least 12 months prior to the experiment. Additional selection criteria included: paresis confined to one side, cortical or subcortical lesions not involving the brainstem or cerebellum, as determined from available neuro-imaging scans or reported in clinical records, absence of severe sensory impairments, severe wasting or contracture and severe cognitive or affective dysfunction, as reported in the registry and ability to provide informed consent. Medications known to suppress central nervous system activity, including alcohol were not allowed. Participants were excluded if they had uncontrolled hypertension. Control participants with no neurological or orthopedic pre-existing conditions were recruited from the community. Participant demographics are listed in Table 1.

**Table 1:** Participant demographics.

**Demographics**						**Clinical assessments**			
**Participant**	**Gender**	**Paresis**	**Age (years)**	**Task completed**	**Time since stroke (months)**	**F-M (out of 34)**	**Berg (out of 60)**	**10 mwt (s) (Comfortable-Fast)**	
S1	Male	Right	58	DH, DA	112	21	55	11.63	6.01
S2	Male	Right	53	DH	43	17	54	10.01	7.58
S4	Male	Left	68	DH, DA	124	18	28	94	82
S5	Female	Right	60	DH, DA	71	18	52	12.95	10.88
S6	Female	Right	64	DH	115	19	53	11.25	8.63
S7	Male	Left	59	DH, DA	46	20	51	8.80	7.43
S9	Male	Right	54	DH, DA	29	19	50	9.57	8.42
S10	Male	Right	59	DH, DA	70	18	40	18.00	13.23
S11	Male	Right	51	DH	56	19	50	11.72	10.16
S13	Male	Right	58	DH	32	21	54	8.58	7.38
S14	Female	Left	59	DH	297	15	51	12.63	10.22
S15	Male	Right	67	DH	96	21	51	13.97	11.62
C2	Male		55	DH				
C3	Male		43	DH, DA				
C4	Female		58	DH, DA				
C5	Male		70	DH				
C6	Male		54	DH				
C7	Female		60	DH				
C8	Female		53	DH, DA				
C9	Male		62	DH, DA				

*Experimental protocols are labeled as DH, dual task hip; DA, dual task ankle. Each lower extremity was tested on a separate visit to the lab. Some participants completed the entire study, while other participants only completed portions of the protocol*.

### Experimental setup

**Figure 1:**
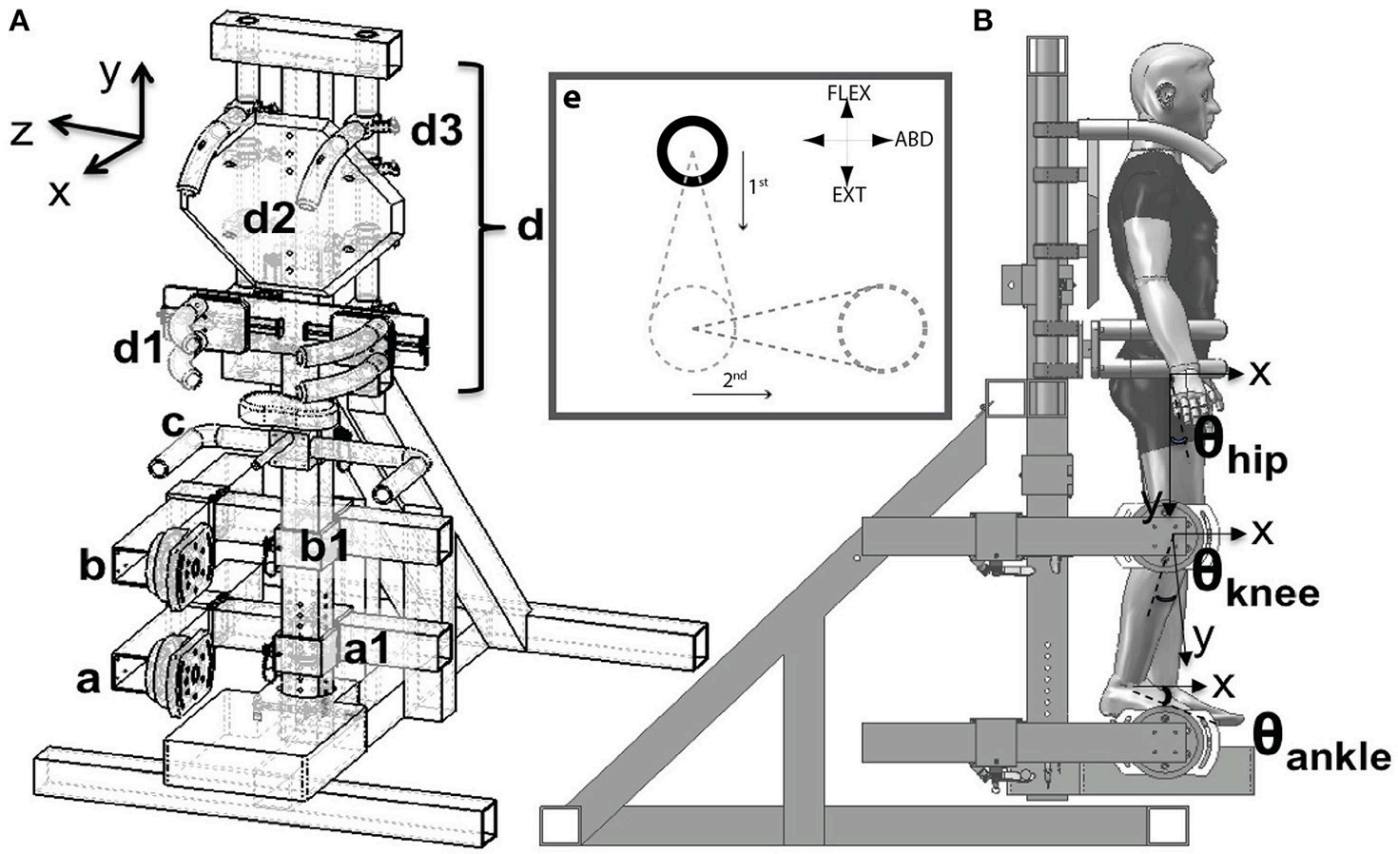
Schematic of the experimental setup. **(Aa,b)** Adjustable foot and thigh placement system and JR3 6DoF sensors. **(c)** Handle bars for accessing the setup. **(d)** Upper body positioning system. **(d1)** Hip clamping mechanism. **(d2)** Back plate. Harness not shown. **(d3)** Shoulder clamps. **(e)**. Visual display. The cursor (solid circle) could move across the entire plane. The dashed circles and lines indicate the target torque and target trajectory. Participants were requested to first match the vertical distance by generating the corresponding hip flexion/extension or ankle plantarflexion torque. Then, while maintaining the vertical position constant, participants were asked to generate the abduction torque necessary to match the horizontal displacement. The hip abduction torque was then quantified as that generated while the vertical level was constant. The display was adjusted for left/right extremities and flexion/extension torques accordingly such that upward displacement indicated flexion and downward extension, displacement to the left was left hip abduction and vice versa for the right extremity. **(B)** Angle descriptions, sagittal plane. The hip flexion angle was measured with respect to the projection of the trunk's gravity vector. The knee flexion angle was measured with respect to the thigh. The ankle angle was measured with respect to the shank. The hip abduction angle (frontal plane), was measured with respect to the gravity vector's direction.

Participants were fitted into the MultiLEIT [Figure 1; see (27)]. Joint angles for the tested leg were set to 20° hip and knee flexion, 0° ankle flexion and 10° hip abduction. The contralateral lower extremity was placed on an elastic sling. Participants' pelvis, trunk and shoulders were rigidly secured into the setup to minimize movement. The MultiLEIT was used to measure torques in the hip flexion/extension, hip abduction/adduction, knee flexion/extension and ankle dorsi/plantarflexion degrees of freedom (DOF).

EMG was measured for all tasks. Differential EMG sensors (Delsys Bagnoli, Delsys Inc., Boston MA) were placed on the muscle belly of the gluteus maximus (GMax, hip extensor), gluteus medius (GMed, hip abductor), adductor longus (AddL, hip adductor), the lateral portion of the quadriceps complex [Rectus Femoris/Vastus Lateralis - RF/VL (39)], vastus medialis (VM, knee extensor), biceps femoris (BF, hip extensor and knee flexor), semimembranosus (SM, hip extensor and knee flexor), medial head of the gastrocnemius (Gastroc, knee flexor and ankle plantarflexor), soleus (Sol, ankle plantarflexor) and tibialis anterior (TA, ankle dorsiflexor). The reference electrode was located on the patella of the tested leg. The skin was prepared by light abrasion and cleansing with alcohol before EMG electrode placement. EMGs were measured only for the leg generating the instructed torques.

### Experimental design

Each extremity of participants post-stroke was tested on separate visits to the lab to limit physical exertion given the requirements of the tasks. The dominant leg of control participants was tested, and this leg was specified as the one used to kick a football. This was the right leg for all participants. Blood pressure and heart rate were measured after each trial to ensure resting levels before starting a new trial. Participants were asked to perform maximum volitional torques (MVTs) in either hip abduction, hip adduction, hip flexion, hip extension, knee flexion, knee extension, ankle dorsiflexion and ankle plantarflexion for a total of 8 tasks. Maximum voluntary torques and maximum EMG recordings were obtained from this portion of the study for normalization purposes, to express differences in MVT and EMG amplitude as a deviation from maximum voluntary activation. Submaximal effort levels were calculated for each participant as 25, 50, and 75% of hip flexion, hip extension and ankle plantarflexion MVTs for the dual DOF tasks.

In the dual DOF tasks participants were instructed to generate hip abduction MVT while generating 25, 50, 75% MVT of either hip flexion (to test whether coupling inside the flexor synergy aided hip abduction torque generation), hip extension or ankle plantarflexion (to determine whether participants can generate torque couples away from the extensor synergy). On the visual display, hip flexion was mapped to displacements in the upward vertical direction. Hip extension or ankle plantarflexion were mapped to displacements in the downward vertical direction. Participants generated the flexion/extension torque first. Once the desired level of flexion/extension torque was achieved, participants were asked to maintain this torque constant, i.e., the vertical position of the cursor was required to remain in the same vertical location on the visual display. Then, participants were asked to generate the largest abduction torque possible which would result in a horizontal displacement of the target. Hip abduction was mapped in the rightward horizontal direction for the right extremity and leftward horizontal direction for the left extremity (Figure 1e). Each task was repeated until the maximum level of hip abduction was achieved consistently and flexion/extension/abduction moments began to decrease due to fatigue.

### Signal processing

Data acquisition was performed using a National Instruments data acquisition unit (PCI 6031E, National Instruments, Austin, Texas). Digital signal processing and data transformation was performed in a custom Matlab graphical user interface (GUI) (Mathworks Inc., Natick, MA). Data were acquired for each trial for 8 s at 1,000 Hz sampling frequency.

Force and torque data were smoothed using a 250 ms moving average window. Joint torques were calculated using Jacobian transformation matrices applied to the smoothed force and torque data measured by the MultiLEIT (27). The hip abduction torque achieved at each level was defined as the maximum torque value obtained while the hip flexion/extension or the ankle plantarflexion was within 10% of the target submaximal torque. Torque data was expressed as a percentage of the MVT for the corresponding DOF measured at the beginning of the experiment.

Hip abduction torque magnitude was normalized by body mass to allow for comparison of the required hip abduction torques between groups and between non-paretic and paretic extremities.

EMG data was baseline corrected, full wave rectified and smoothed using a 250 ms moving average window. The mean EMG amplitude corresponding to the MVT was identified and averaged for the 50 ms leading to the time-point of MVT for each task. EMGs were normalized to the peak maximum activation obtained for the task in which the muscle was expected to provide the greatest joint torque contribution based on anatomical action and moment arm (40) and expressed as %MVT-EMG. The tasks used to normalize the EMGs were hip abduction for GMed, hip adduction for AddL, knee extension for RF/VL and VM, knee flexion for BF, SM, Gastroc, hip extension for GMax, ankle plantarflexion for Sol and ankle dorsiflexion for TA.

### Statistical analysis

All statistical analyses were run in SPSS (version 21, IBM Corp.). Independent variables were defined as group (control, stroke) lower extremity (control, non-paretic, paretic) and hip flexion/extension or ankle plantarflexion level (25, 50, or 75%MVT). Hip abduction/adduction torque was defined as the dependent variable. Data was tested for normality using Q-Q plots. Homogeneity of variance was tested using Levene's Statistic. A linear mixed model was used with group, lower extremity and level and with extremity nested within group as fixed factors and participant as a random factor. Hip abduction MVT magnitude was included as a covariate to account for biases induced by hip abduction torque magnitude, i.e., given that participants-post stroke generate hip abduction MVTs that are about 50% less than those generated by healthy individuals (25), the magnitude of the torque combinations differed between healthy and post-stroke individuals. Statistical tests aimed to determine differences in volitional hip abduction MVT generated during submaximal hip flexion, hip extension and plantarflexion. Post-hoc analyses were run using the Sidak adjustment for multiple comparisons. EMGs were analyzed in the same manner, to determine differences in muscle activation during the dual tasks between across paretic, non-paretic and control lower extremities.

The relationship between hip abduction/adduction and hip extension was fit with a linear regression for each lower extremity tested (control participants and both extremities of participants post-stroke). 95% confidence intervals for the regression lines were calculated for each percentage of torque. The x-intercept and y-intercepts for each regression line was calculated; particularly, the x-intercept indicates the approximate extension level participants could generate before spontaneous hip adduction (as defined in the extensor synergy) would occur i.e., a net abduction/adduction torque of 0 Nm/MVT. We used the slope of the relationship as a proxy for the strength of the extensor synergy coupling.

## Results

### MVT values across groups

In agreement with previous results (25), we measured significant hip abduction weakness in the paretic extremity of participants post-stroke. On average, normalized mean peak hip abduction torque (MVT/mass) for the control group was 0.660 ± 0.098 Nm/kg. In the non-paretic extremity, mean peak torque accounted to 0.725 ± 0.071 Nm/kg and in the paretic extremity, peak hip abduction was on average 0.261 ± 0.067 Nm/kg. The maximum hip abduction torques that participants post-stroke generated with the paretic lower extremity were significantly smaller (*F* = 48.142, *p* < 0.001) compared to the non-paretic (*p* < 0.001) and to the control (*p* < 0.001) lower extremities. For each participant, these maximum hip abduction torques were the target they needed to accomplish during the dual task.

### Submaximal hip flexion + hip abduction MVT

**Figure 2:**
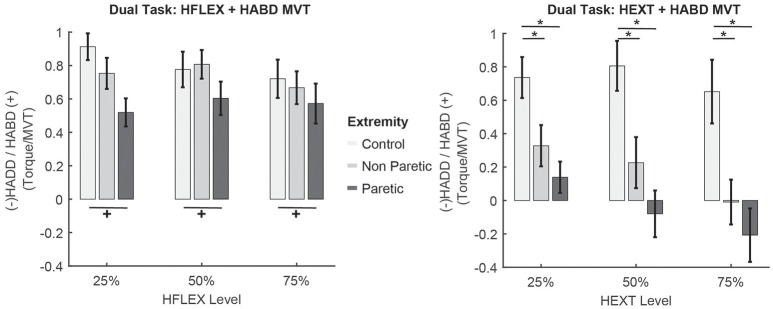
Results for the combined submaximal hip flexion + hip abduction MVT task and hip extension + hip abduction MVT task. x-axis indicates the percentage of hip flexion/extension and the y-axis indicates the volitional hip abduction or spontaneous hip adduction generated, normalized by hip abduction MVT. Percentages reported in the main text correspond to this normalized value × 100%. Off-white = control, gray = non-paretic, black = paretic. ^+^*p* < 0.05 Sidak post-hoc significant differences across lower extremities in the % hip abduction torque achieved. ^*^p < 0.05 Sidak post-hoc significant differences across levels and lower extremities in the % hip abduction torque achieved. Error bars show standard errors of the mean.

Given post-stroke hip abduction weakness, we expected that instructed coupling within the flexor synergy would aid generation of hip abduction torques above MVT. Mixed model analyses did not return a significant main effect of group (control, stroke, *F* = 0.488, *p* = 0.697) or hip flexion level (*F* = 0.465 *p* = 0.630) on hip abduction torque. However, we observed a significant effect of lower extremity on hip abduction magnitude (*F* = 5.474, *p* = 0.022), with the paretic lower extremity generating lower levels of hip abduction compared to the control (*p* = 0.025) and marginally lower hip abduction than the non-paretic (*p* = 0.064) lower extremity (Figure 2). Contrary to our expectations, combining hip flexion with hip abduction did not aid participants post-stroke increase hip abduction torques magnitude with their paretic extremity.

#### EMG activation during submaximal hip flexion + hip abduction

No significant differences in GMed activation were observed between lower extremities (*F* = 0.122, *p* = 0.728), across levels of hip flexion (*F* = 0.164, *p* = 0.849) or between groups (*F* = 2.124, *p* = 0.149). AddL activation did not change across levels of hip flexion (no significant effect of level, *F* = 0.989, *p* = 0.377) but was marginally different between lower extremities (*F* = 3.531, *p* = 0.064) and between groups (*F* = 5.658, *p* = 0.020), with overall greater AddL co-activation in the paretic extremity than in the controls' extremity (*p* = 0.011) and overall greater activation in stroke participants than controls (*p* = 0.020). These results indicate that coupling with hip flexion does not increase drive to hip abductors which could aid increase hip abduction torque.

### Submaximal hip extension + hip abduction MVT

The capacity to generate hip abduction during hip extension was impaired in both extremities of participants post-stroke. Results from the statistical analyses showed significant main effects of group (*F* = 33.723, *p* < 0.001), lower extremity (*F* = 5.012, *p* = 0.028) and level of hip extension (*F* = 3.375, *p* = 0.039) on the amount of hip abduction generated. At 25% hip extension, control participants generated 74 ± 30% hip abduction MVT, whereas participants post-stroke generated 33 ± 39% hip abduction MVT with their non-paretic extremity (*p* < 0.001 compared to control participants) and 14 ± 34% hip abduction MVT with their paretic extremity (*p* < 0.001 compared to control participants and *p* = 0.082 compared to the non-paretic extremity). At 50% hip extension, control participants generated 80 ± 37% hip abduction MVT, whereas participants post-stroke generated 22 ± 48% hip abduction MVT with their non-paretic extremity (*p* = 0.021 compared to control participants) and −8 ± 48% hip abduction MVT with their paretic extremity (the negative sign indicates net adduction, *p* < 0.001 compared to control participants and *p* = 0.146 compared to the non-paretic extremity). Finally, at 75% hip extension, control participants generated 65 ± 47% hip abduction MVT, whereas participants post-stroke generated 0 ± 42% hip abduction MVT with their non-paretic extremity (*p* < 0.001 compared to control participants) and −23 ± 56% hip abduction MVT with their paretic extremity (the negative sign indicates net adduction, *p* = 0.001 compared to control participants and *p* = 0.313 compared to the non-paretic extremity). To summarize, in the non-paretic extremity, as the level of voluntary hip extension torque increased, participants' capacity to generate hip abduction decreased. In the paretic extremity, the voluntary hip abduction torque generated was not sufficient to balance the spontaneous hip adduction torque, resulting in net hip adduction at the 50 and 75% MVT levels, whereas control participants were capable of generating levels of hip abduction close to MVT for all hip extension levels (Figure 2).

#### EMG activation during submaximal hip extension + hip abduction MVT

Activation of GMed differed between participants post-stroke and controls. We measured a significant main effect of group (*F* = 6.700, *p* = 0.012) and lower extremity (*F* = 3.485, *p* = 0.038) on GMed activation. However, activation did not vary across levels (*F* = 0.371, *p* = 0.691). Overall, participants post-stroke generated a greater %MVT-EMG than control participants (*p* = 0.012) and this was mostly due to increased activation in their paretic extremity compared to controls (*p* = 0.038, no differences were observed compared to the non-paretic leg, *p* = 0.972). Hip extension level did not have a significant main effect on AddL EMG (*F* = 0.192, *p* = 0.826). However, a significant effect of lower extremity was quantified (*F* = 4.106, *p* = 0.046) as well as a significant difference between controls and participants post-stroke (*F* = 17.202, *p* < 0.001). Overall, participants post-stroke had greater co-activation of their AddL compared to controls (*p* < 0.001), both in the paretic (*p* < 0.001) and non-paretic (*p* = 0.019) extremities. No differences in the paretic vs. the non-paretic leg were measured (*p* = 0.133). Despite the apparent increased activation of the GMed, participants post-stroke were not able to overcome AddL co-activation to generate net hip abduction torques and satisfy the requirements of the experimental task.

In participants post-stroke, we observed a persistent extensor coactivation across all levels of hip extension. Overall, participants post-stroke showed higher co-activation of the VM compared to controls (*F* = 8.893, *p* = 0.004). The Gastroc muscle was more active in participants post-stroke than in controls (*F* = 13.089, *p* < 0.001), with marked co-activation (*F* = 6.580, *p* = 0.002) of both paretic (*p* = 0.004) and non-paretic (*p* = 0.007) lower extremities. Sol activation was also increased post-stroke (*F* = 18.971, *p* < 0.001) and differed between lower extremities (*F* = 12.691, *p* < 0.001) with the paretic lower extremity averaging greater Sol activation compared to controls (*p* < 0.001) and compared to the non-paretic extremity (*p* = 0.068). The non-paretic Sol was also more active than the control Sol (*p* = 0.016).

### Sub-maximal ankle plantarflexion + hip abduction MVT

**Figure 3:**
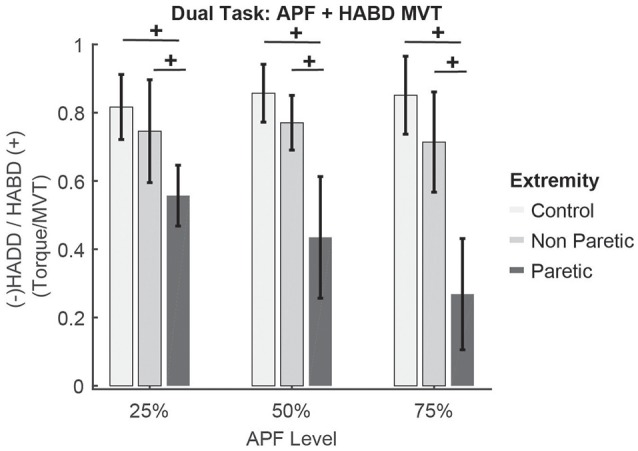
Results for the combined submaximal ankle plantarflexion + hip abduction MVT task. x-axis indicates the percentage of ankle plantarflexion. y-axis indicates the volitional hip abduction or spontaneous hip adduction generated, normalized by hip abduction MVT. Percentages reported in the main text correspond to this normalized value × 100%. ^+^*p* < 0.05 Sidak post-hoc significant differences across lower extremities in the % hip abduction torque achieved. No differences between levels of plantarflexion were observed. Error bars show standard error of the mean. Colors are those defined in Figure 2.

No differences in hip abduction torque during ankle plantarflexion were quantified across levels (*F* = 0.227, *p* = 0.798; Figure 3). However, a significant main effect of group (*F* = 5.713, *p* = 0.021) and lower extremity was seen (*F* = 14.598, *p* < 0.001), with participants post-stroke generating overall lower hip abduction torques than controls. Specifically, participants generated less hip abduction with the paretic extremity compared to their non-paretic extremity (*p* = 0.001) and compared to control participants (*p* = 0.001).

During submaximal plantarflexion + hip abduction, the amount of spontaneous hip extension increased as plantarflexion level increased (main effect of level, *F* = 3.263, *p* = 0.048). However, no differences were seen between post-stroke and control participants (*F* = 2.142, *p* = 0.151), but a trend was seen between lower extremities (*F* = 3.392, *p* = 0.072) with control participants generating lower coupled hip extension.

#### EMG activation during submaximal ankle plantarflexion + hip abduction MVT

Activation of GMed during ankle plantarflexion + hip abduction was not affected by group (*F* = 0.024, *p* = 0.877), lower extremity (*F* = 2.484, *p* = 0.124), or level (*F* = 0.031, *p* = 0.969). In contrast, greater activation of AddL was observed in participants post-stroke (main effect of group *F* = 12.797, *p* = 0.001) and between control, paretic and non-paretic lower extremities (*F* = 7.667, *p* = 0.002), but not across plantarflexion levels (*F* = 0.093, *p* = 0.911). AddL activation was greater in the paretic extremity compared to control participants (*p* = 0.001).

### Strength of extension/adduction coupling

**Figure 4:**
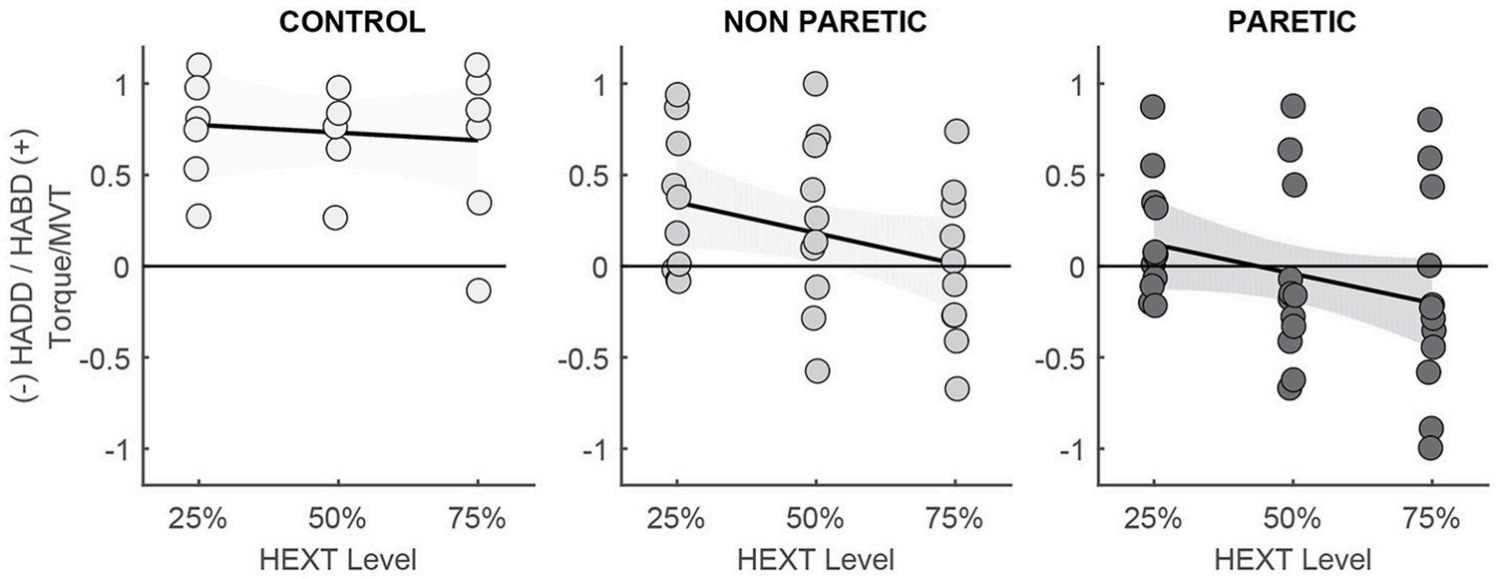
Individual data and linear regression lines to illustrate the relationship between hip extension/adduction. The flat regression line and lack of significant correlation in control participants indicates independence between hip extension and abduction. The relationship between hip extension and abduction/adduction is seen in the paretic extremity and to a lesser degree in the non-paretic extremity.

Linear regression analyses in the lower extremities of control and post-stroke participants indicated differences in coupling strength and the maximum amount of hip extension allowed before hip abduction could no longer be generated (Figure 4). Linear regression equations in control (C), non-paretic (NP), and paretic (P) extremities were:

$$\begin{array}{l} HipAbdAdd_{C}=0.816+0.169*HipExt;\\ \text{ R}=0.097,p=0.702.\text{y}-\text{intercept significantly}\\ \text{ different from zero }\left(p<0.005\right).\\ HipAbdAdd_{NP}=0.519+0.675*HipExt;\\ \text{ R}=0.316,p=0.089.\text{y}-\text{intercept significantly}\\ \text{ different from zero }\left(p<0.005\right).\\ HipAbdAdd_{P}=0.289+0.792*HipExt;\\ \text{ R}=0.346*,p=0.008.\text{Intercept not significantly}\\ \text{ different from zero}. \end{array}$$

For the linear regression, all torques were expressed as % MVT. We assigned negative values to hip extension (but plotted as positive for ease of visualization) and adduction while hip abduction was assigned positive values (Figure 4). No significant correlation between hip extension and abduction was observed in the lower extremity of control participants. In the non-paretic extremity, there is a trend in the association between hip extension torque and hip abduction. Based on the linear regression equation, the amount of predicted hip extension torque that would result in spontaneous coupling with hip adduction would be 77% MVT in the non-paretic extremity of participants post-stroke. The correlation between hip extension and abduction/adduction is significant in the paretic extremity of participants post-stroke, such that an increase in hip extension increases hip adduction, i.e., 34.6% of the variability in hip abduction/adduction torque is due to coupling with hip extension. Based on the linear regression equation, the amount of hip extension needed to overcome voluntary hip abduction would account to 36.5% MVT. As seen in Figure 4, the majority of participants were unable to generate hip abduction during levels of hip extension greater than 25%. These results indicate that the capacity to generate hip abduction torques depends on the torques generated in the other degrees-of-freedom and the neural drive required to generate these torques. The three participants who were capable of generating hip abduction torques during hip extension generated hip abduction MVTs with the paretic extremity that were around 25% of the magnitude generated by their non-paretic extremity. Therefore, even though they appear to combine hip abduction with hip extension, their hip abduction MVTs are reduced compared to the torques required during locomotion [4, 33, and 20 Nm respectively compared to approximately 160 Nm in healthy individuals and in the non-paretic extremity, as previously reported in reference (25)]. These participants were accounted for in the mixed model analysis by using hip abduction magnitude as a covariate.

## Discussion

In the present study, we sought to determine whether the capacity to generate hip abduction torques in participants post-stroke is affected by the torques generated at the hip or ankle joints. Results from the present study indicate an abnormal, progressive inability to generate simultaneous hip extension/abduction torques in both lower extremities of participants post-stroke, supporting our hypothesis that participants post-stroke are constrained to the extensor synergy during hip extension tasks. These limitations affect stroke survivors by reducing and potentially eliminating their ability to generate volitional net hip abduction with concurrent hip extension in the paretic lower extremity, and instead involuntarily coupling hip adduction and hip extension. Interestingly, these kinetic constraints also affect the non-paretic extremity; synergy-induced hip adduction seems to limit the ability to generate hip abduction torques to a point where no net abduction torques can be measured in the non-paretic leg. Thus, for the first time we quantified bilateral changes in the voluntary control of the lower extremity, and provide evidence to support the existence of bilateral expression of abnormal limb synergies in hemiparetic stroke. This is in contrast to the upper extremity, where abnormal joint torque coupling patterns, in accordance to the clinical synergies are only present in the paretic arm (20–24).

### The capacity to generate voluntary hip abduction torques is not enhanced by hip flexion

The purpose of this experimental task was to test whether instructing participants post-stroke to use a coupling strategy such as that described clinically in the flexor synergy [coupling of flexion/abduction—(28, 29]) could help increase mechanical coupling and descending drive to increase the hip abduction torques beyond MVT, i.e., given their weakness when generating MVTs in hip abduction, can they increase hip abduction when it is generated as a coupled torque. Our results did not support this hypothesis, and in fact, the relative hip abduction torque generated in the paretic lower extremity was lower than in controls. These results might be explained by the increased co-activation of the AddL muscle in the paretic extremity, which serves as both hip flexor and hip adductor. These findings are an indication that the flexor synergy may not be elicited in the lower extremity during upright posture (25) possibly due to an overall extension bias (41). Historically, lower extremity synergies have been described in the supine posture (28), which provides different postural drive and mechanical requirements. In an upright posture, combining hip flexion and abduction does not serve to enhance already weakened hip abduction torques (25).

### After stroke, the capacity to generate voluntary hip abduction torques decreases when generating hip extension

Our results indicate that the capacity to generate hip abduction in healthy individuals is not affected by hip extension requirements. Control participants generated on average close to 70% of their maximum hip abduction torque while maintaining constant submaximal levels of hip extension. Reductions from MVT in healthy participants may be due to the difficulty of the task, given that they are instructed to maintain hip extension within 10% of the target level before generating maximal hip abduction. Previous research by our group quantified spontaneous coupling of hip adduction with hip extension in control participants when generating maximal and submaximal hip extension torques (25). The present results indicate that when instructed, control participants can voluntarily drive their hip musculature in patterns that allows them to combine hip extension with hip abduction, away from the hip extension/adduction coupling observed in single degree of freedom, maximum hip extension torques (25).

In contrast, in participants post-stroke, the ability to couple hip extension and abduction torques was limited in the non-paretic extremity and absent in the paretic extremity. In the paretic lower extremity, a combination of hip abduction weakness and significant adductor coactivation (25), as reported previously, impeded participants from combining hip extension with hip abduction torques. In fact, EMG findings show that despite apparently greater activation of GMed musculature, participants did not generate hip abduction torques in their paretic extremity to overcome hip adduction. A potential explanation of these impairments is increased reliance on bilateral brainstem pathways such as the vestibulospinal tract (42, 43) supported by evidence from animal models, where combined ablation of the pyramidal tracts and of the reticulospinal tracts is associated with increased resistance of hip adductor musculature (44). In the non-paretic extremity, preserved hip abduction strength may allow participants to overcome spontaneous coupling up to a certain amount of hip extension until increased bilateral drive from brain stem pathways overcomes voluntary corticospinal drive (25).

### Generation of distal plantarflexion does not influence the capacity to generate voluntary hip abduction torques

No significant differences between control participants and the paretic or non-paretic lower extremity were quantified in the hip abduction torque when generating 25 and 50% ankle plantarflexion combined with hip abduction MVT. Only at 75% ankle plantarflexion, was there a significant decrease in paretic hip abduction torque compared to both control and non-paretic extremities. Access to remaining corticospinal resources and decreased dependence on brainstem pathways of more distal muscles that control the ankle plantarflexion task can explain why participants can “overcome” the extensor synergy. Results also indicate that the coupling is stronger when elicited proximally (hip) than when elicited distally (ankle), as seen in the upper extremity (45, 46). Participants did not show any difference in behavior from control participants with their non-paretic extremity, indicating that this task was driven by unaffected, contra-lesional corticospinal pathways.

### Increased reliance on bilateral postural pathways may explain decreased hip abduction post-stroke

The lower extremity is highly innervated by brainstem postural pathways, specifically vestibulospinal and reticulospinal pathways, due to its primordial postural role in standing and upright balance (41). Changes in the descending control of the lower extremity observed in both the paretic and non-paretic extremities can be explained by reliance on unimpaired and bilateral bulbospinal pathways after the loss of cortical projections due to the stroke-induced lesion (19, 20). Brainstem innervation of the lower extremity is mostly bilateral in nature, which may explain the bilateral effects on the inability to generate certain torque coupling patterns examined in this study. Specifically, in the cat, the hind limbs receive innervation from the ipsilateral, lateral vestibulospinal tract which coordinates and adjusts the activity and tone of the ipsilateral extensor motor neurons during rest and locomotion (47). We hypothesize that these vestibulospinal pathways may be enhanced after stroke, especially in a task where muscles need to be posturally active, such as in standing similarly to our experimental setup. Therefore, under our experimental posture, vestibular pathways may have a significant effect on lower extremity activation driving extension and adduction in a multi-degree-of-freedom pattern. Also, studies using galvanic vestibular stimulation in humans, have shown increased activation of proximal bilateral hip extensors gluteus maximus and biceps femoris (48). This bilateral effect of vestibular drive is thought to occur via commissural interneurons that can activate contralateral muscles (30, 41, 49). Furthermore, ablation of the vestibulospinal tract in non-human primates (44) generated bias toward hip flexion and abduction, knee extension and ankle dorsiflexion, and abolished extensor tone (50). These findings support our hypothesis that increased reliance on brainstem pathways, specifically the vestibulospinal tract, explain the bilateral bias toward extension and adduction.

In addition to the effects of the vestibulospinal tract, the reticulospinal tract may also contribute to the joint torque limitations quantified in the present study. Among the many functions controlled by this system, this pathway elicits excitatory post-synaptic potentials of extensor motor neurons (51). In human control of the lower extremity, the role of the reticulospinal pathway has remained relatively inaccessible to experimentation. However, studies using proxies of reticular function including the bilateral startle response (31, 52) have attempted to understand the role of the reticulospinal system. The startle response is posture specific: when a startle is presented with the participant in a sitting posture, a flexion pattern is elicited. In contrast, when participants are startled in standing, the reticulospinal tract will generate a response that accomplishes stance with maximal postural stability by exciting extensor muscles (31). Thus suggesting that reticular activation of extensor muscles occurs only when the muscle is posturally relevant, such as in standing (52, 53) and may also explain the extension bias observed in this study in the lower extremities of post-stroke participants. Therefore, we infer that the extension constraints in the lower extremity that we quantified in upright posture may be driven through vestibular- and reticular- pathways to the lower extremity. This drive may be stronger than that from remaining corticofugal pathways and therefore the kinetic coupling arises when the drive from these bulbospinal pathways overcomes the drive from remaining cortical resources.

### Clinical implications

Results from the present study indicate that in a functionally relevant posture, such as upright stance, post-stroke participants will have limited access to hip abduction torques. These kinetic limitations may compromise standing posture and balance where hip extension/abduction coupling patterns are required preserve balance and to be able to overcome external perturbations. The current findings may help explain the increased risk for falls (15, 54, 55) that can result in hip fractures on the paretic side (56). Functional implications of this coupling may also be seen in tasks such as gait initiation, which requires the combination of hip extension to generate forward momentum and hip abduction to shift the center of pressure between extremities and stabilize the body weight over the supporting leg (57–59). Given our isometric protocol, we cannot extrapolate these findings to gait, where the dynamics of the task as well as muscle lengths may differ, and therefore additional studies are needed to determine if the increased occurrence of falls during walking (54) is due to the abnormal extension/adduction coupling. The impact of the hip extension/adduction coupling can be addressed through physical rehabilitation interventions that focus on strengthening hip abduction while progressively increasing hip extension, such as in sit-to-stand, gait initiation, side-stepping and other functional activities, with the ultimate goal to improve the ability of stroke survivors to generate joint torque coupling patterns outside of the constraining abnormal coupling pattern.

## Ethics statement

This study was carried out in accordance with the recommendations of Northwestern University IRB with written informed consent from all subjects. All subjects gave written informed consent in accordance with the Declaration of Helsinki. The protocol was approved by the Northwestern University IRB.

## Author contributions

NS, AA, and JD: study design. NS and RL-R: data acquisition. NS: data analysis and manuscript preparation. AA and JD: manuscript editing. All authors contributed to manuscript revision, read and approved the submitted version.

### Conflict of interest statement

The authors declare that the research was conducted in the absence of any commercial or financial relationships that could be construed as a potential conflict of interest.
